# Reply to Di Ciaula, A.; Portincasa, P. Comment on “Huang et al. Accuracy and Agreement of a 45 mg Versus a 75 mg ^13^C-Urea Breath Test for the Diagnosis of *Helicobacter pylori* Infection in Adults: A Randomized Crossover Trial. *Diagnostics* 2026, *16*, 1567”

**DOI:** 10.3390/diagnostics16182923

**Published:** 2026-09-10

**Authors:** Jun Huang, Yaohong Xie, Jian Huang, Bingyun Lu, Ye Chen

**Affiliations:** 1 Guangdong Provincial Key Laboratory of Gastroenterology, Department of Gastroenterology, Nanfang Hospital, Southern Medical University, Guangzhou 510000, China; 2 Integrative Microecology Clinical Center, Shenzhen Key Laboratory of Gastrointestinal Microbiota and Disease, Shenzhen Clinical Research Center for Digestive Disease, Shenzhen Technology Research Center of Gut Microbiota Transplantation, Department of Gastroenterology, Shenzhen Hospital, Southern Medical University, Shenzhen 518000, China

We thank Di Ciaula and Portincasa for their thoughtful and constructive Comment on our randomized crossover trial comparing two ^13^C-urea breath test (UBT) protocols [1,2]. Their observations delineate important methodological boundaries for interpreting our findings. We welcome this opportunity to clarify the study objective, state the limitations more explicitly, and refine our conclusions. In particular, diagnostic agreement, diagnostic accuracy, the absence of a statistically significant difference, equivalence, non-inferiority, and interchangeability are distinct concepts and should not be conflated. Agreement indicates how often two tests assign the same classification to the same individuals; it does not establish whether either classification is correct. Accuracy requires comparison with an appropriate reference standard, whereas equivalence and non-inferiority require prospectively specified estimands, margins, and analytical frameworks. Similarly, failure to detect a statistically significant difference does not constitute affirmative evidence of equal clinical performance.

We agree that the composite reference standard used in the secondary diagnostic-accuracy analyses introduces risks of incorporation bias and circularity. Of the 431 participants included in the per-protocol analysis, only the 42 with discordant 30-min UBT classifications underwent endoscopy, rapid urease testing, and histologic assessment, with immunohistochemistry used when required. For the remaining 389 participants, who had concordant UBT results, the shared classification of the two index tests was assumed to represent the true infection status. Because the index tests thus contributed to the reference classification for most participants, sensitivity, specificity, predictive values, and AUC may have been overestimated. This limitation also precludes the use of those estimates to establish formal equivalence or non-inferiority.

The primary endpoint was diagnostic agreement between the 45-mg and 75-mg protocols at 30 min, rather than absolute diagnostic accuracy against an independent reference standard in every participant [2]. This choice reflected the ethical and practical constraints on performing endoscopy in every participant, particularly among individuals tested for screening or in settings where endoscopy was not otherwise indicated. The paired crossover design was informative for within-participant concordance but could not overcome the absence of independent verification when absolute accuracy was estimated. The composite reference standard was used only for the secondary accuracy analyses and does not eliminate the limitations associated with partial verification. These accuracy findings should therefore be regarded as supportive and exploratory. Further validation should apply an independent reference standard to all participants or to a prespecified random sample, with analyses that appropriately account for verification and sampling.

We also agree that the study did not isolate the effect of urea dose. Rather, it compared two complete clinical testing protocols as used in practice. The 45-mg protocol used The Kit for [^13^C]-Urea Breath Test, a 45-mg tablet without citric acid (Beijing Haiderun Pharmaceutical Group Co., Ltd., Beijing, China; Chinese National Drug Approval No. H20174047). The 75-mg protocol used the Urea [^13^C] Breath Test Kit, supplied as 5-g granules containing 75 mg of ^13^C-urea and citric acid (Beijing Huagen Anbang Technology Co., Ltd., Beijing, China, marketing authorization holder; Chinese National Drug Approval No. H20061169). No separate proprietary trade names are listed in the approval information available to us; we therefore report the approved generic kit names. The protocols differed simultaneously in urea dose, formulation, and gastric acidification conditions. Citric acid may influence DOB through intragastric acidification, gastric emptying, substrate distribution, and changes in the duration or extent of contact between urea and bacterial urease [3,4,5,6,7,8]. The observed differences therefore cannot be attributed to the amount of ^13^C-urea alone, and our study should not be interpreted as disproving a physiological or diagnostic role for citric acid. Even if the complete 45-mg protocol performs acceptably in some settings, the present data do not establish which component of the protocol accounts for that performance. The supplementary analysis indicated that continuous DOB values were generally lower with the 45-mg protocol, although categorical classifications showed substantial overall agreement. This observation is exploratory and hypothesis-generating and does not prove a causal dose or formulation mechanism. Ideally, future studies should standardize the formulation, excipients, and acidification conditions and vary only the urea dose.

The same-day crossover design is another legitimate concern. The two tests were separated by at least 2 h (mean interval, 153.17 min), and a new baseline breath sample was obtained before each intervention [2]. The second-period baseline DOB data are complete and have undergone quality control. Agreement was similar between the randomized sequences: 90.3% with κ = 0.776 in Sequence A (45 mg followed by 75 mg) and 90.2% with κ = 0.754 in Sequence B (75 mg followed by 45 mg). These findings show no obvious order-related difference, but they do not exclude residual isotope or other carryover effects. The availability of quality-controlled baseline data and sequence-stratified agreement provides some reassurance but is not a substitute for a model designed to estimate carryover, period, and sequence effects. No formal carryover analysis was prespecified or reported; we therefore do not claim that a post hoc assessment resolves this design limitation. Future crossover studies should preferably administer the protocols on different days or prespecify analyses of carryover, period, and sequence effects, including evaluation of the baseline obtained before the second period.

We appreciate the concern regarding data-derived cutoffs. The categorical agreement analysis must be distinguished from the ROC-based accuracy analyses. For categorical agreement, each DOB measurement was classified according to a fixed clinical rule: <5.0‰ was negative and ≥5.0‰ was positive. In contrast, the reported ROC thresholds were selected and evaluated in the same dataset by maximizing the Youden index and were assessed against the same composite reference standard [2]. The latter approach is susceptible to optimism and overfitting and may overstate diagnostic performance. The ROC-derived thresholds are secondary, exploratory findings and should not be regarded as externally validated clinical cutoffs. Future studies should prespecify manufacturer-defined or independently validated thresholds and evaluate them in a separate cohort. More broadly, the absence of a statistically significant difference between the reported AUCs (0.953 and 0.966; *p* = 0.50) does not, by itself, demonstrate equivalence, formal non-inferiority, or clinical interchangeability.

Clinical heterogeneity within the cohort also limits inference. The study included 336 participants undergoing initial diagnosis and 95 undergoing post-eradication confirmation [2]. These populations may differ in bacterial burden, urease activity, and DOB distribution. In the post-eradication setting, a low organism burden and borderline DOB values may increase the risk that a lower-signal protocol yields false-negative results. We did not perform a diagnostic-accuracy analysis with adequate power after stratification by clinical indication, and the aggregate findings do not resolve this issue. The present study therefore does not support preferential use of the 45-mg protocol to confirm eradication. Future studies should evaluate treatment-naïve and post-eradication populations in separate, adequately powered cohorts, using reference standards appropriate to each indication.

The 42 discordant cases require particularly cautious interpretation. Endoscopic reference testing classified 15 as *H. pylori*-positive and 27 as *H. pylori*-negative. Among the 15 positive cases, the 75-mg protocol correctly identified 11 and the 45-mg protocol correctly identified 4; among the 27 negative cases, the 45-mg protocol correctly identified 16 and the 75-mg protocol correctly identified 11 [2]. Because this subgroup was selected on the basis of disagreement between the index tests, it cannot be used to estimate population-level sensitivity, specificity, or overall superiority. Statistical comparisons within this outcome-selected subgroup do not make it representative of the full cohort. Nevertheless, the pattern suggests potentially different operating characteristics: the 75-mg protocol may be more likely to classify borderline or low-burden infections as positive, whereas the 45-mg protocol may have a greater tendency toward negative classification. These findings do not support complete interchangeability across all clinical settings and should be considered hypothesis-generating.

In summary, the most robust finding of our study is the substantial diagnostic agreement at 30 min between the two complete ^13^C-UBT protocols. The secondary accuracy analyses support further evaluation of the 45-mg protocol but do not prove that the protocols are equivalent, definitively non-inferior, interchangeable, or suitable for widespread replacement across clinical indications. Future studies should apply an independent reference standard to all participants or to a prespecified representative sample; isolate urea dose from formulation, excipient, and acidification effects; administer the tests on different days or prespecify analyses of crossover effects; use externally established cutoffs; and stratify enrollment and analysis by initial diagnosis versus post-eradication confirmation. We thank Di Ciaula and Portincasa for helping us define these priorities and place our original conclusions on an appropriately cautious footing.

## Data Availability

No new data were created or analyzed in this reply. Data sharing is not applicable to this article.
